# DNA-free RNA isolation protocols for *Arabidopsis thaliana*, including seeds and siliques

**DOI:** 10.1186/1756-0500-1-93

**Published:** 2008-10-20

**Authors:** Luis Oñate-Sánchez, Jesús Vicente-Carbajosa

**Affiliations:** 1 Centro de Biotecnología y Genómica de Plantas (UPM-INIA), Campus Montegancedo, Universidad Politécnica de Madrid (M-40, km 38), 28223-Pozuelo de Alarcón (Madrid), Spain

## Abstract

**Background:**

High throughput applications of the reverse transcriptase quantitative PCR (RT-qPCR) for quantification of gene expression demand straightforward procedures to isolate and analyze a considerable number of DNA-free RNA samples. Published protocols are labour intensive, use toxic organic chemicals and need a DNase digestion once pure RNAs have been isolated. In addition, for some tissues, the amount of starting material may be limiting. The convenience of commercial kits is often prohibitive when handling large number of samples.

**Findings:**

We have established protocols to isolate DNA-free RNA from *Arabidopsis thaliana *tissues ready for RT-qPCR applications. Simple non-toxic buffers were used for RNA isolation from Arabidopsis tissues with the exception of seeds and siliques, which required the use of organic extractions. The protocols were designed to minimize the number of steps, labour time and the amount of starting tissue to as little as 10–20 mg without affecting RNA quality. In both protocols genomic DNA (gDNA) can be efficiently removed from RNA samples before the final alcohol precipitation step, saving extra purification steps before cDNA synthesis. The expression kinetics of previously characterized genes confirmed the robustness of the procedures.

**Conclusion:**

Here, we present two protocols to isolate DNA-free RNA from Arabidopsis tissues ready for RT-qPCR applications that significantly improve existing ones by reducing labour time and the use of organic extractions. Accessibility to these protocols is ensured by its simplicity and the low cost of the materials used.

## Background

Reverse transcriptase quantitative PCR (RT-qPCR) is a very useful and sensitive technique to quantitate changes in gene expression. Increasing number of labs routinely use the agarose-based or/and real-time versions of the technique. A significant number of replicates are required to ensure significance of the results, which adds a high-throughput component to the RT-qPCR. These requirements impose the need to have simplified protocols to reduce labour and costs without compromising RNA integrity or yield.

Since *Arabidopsis thaliana *is a model organism for plant biologists, a number of RNA extraction protocols have been developed. Although there are several commercial kits available, the cost can be prohibitive when processing large numbers of samples. Published protocols use organic extractions with toxic chemicals, which involve the use of fume hoods to diminish health risks and lengthen the procedure [1-5]. Moreover, none of these protocols reduce genomic DNA (gDNA) contamination enough below PCR-detection levels. Digestion with DNase removes traces of DNA and is compulsory if the RNA samples are devoted to RT-qPCR. DNase digestion after the final RNA precipitation step involves adding extra salts and protein to the sample and, since they can affect the efficiency of the cDNA synthesis, additional purification steps are required. The amount of starting tissue is another factor to consider, especially when enough sample quantities are difficult to obtain and to speed up work by fitting volumes into standard microfuge tubes (SMTs). Polysaccharides and other compounds frequently contaminate RNA samples from seeds and siliques [2] and protocols suitable for RNA isolation from other tissues have to be adapted. Although high-salt extraction buffers have been used to get rid of those contaminants [1-3,5] large RNAs may become insoluble in these buffers, therefore decreasing the complexity and information contained in the samples.

Here we report improved RNA isolation protocols for the model plant *Arabidopsis thaliana *that address the problems mentioned above. Both protocols start with small amounts of tissue and buffer volumes to fit the SMT format, and incorporate a DNase digestion before the final purification steps.

## Results

### RNA extraction from Arabidopsis tissues other than seeds and siliques

**Figure 1:**
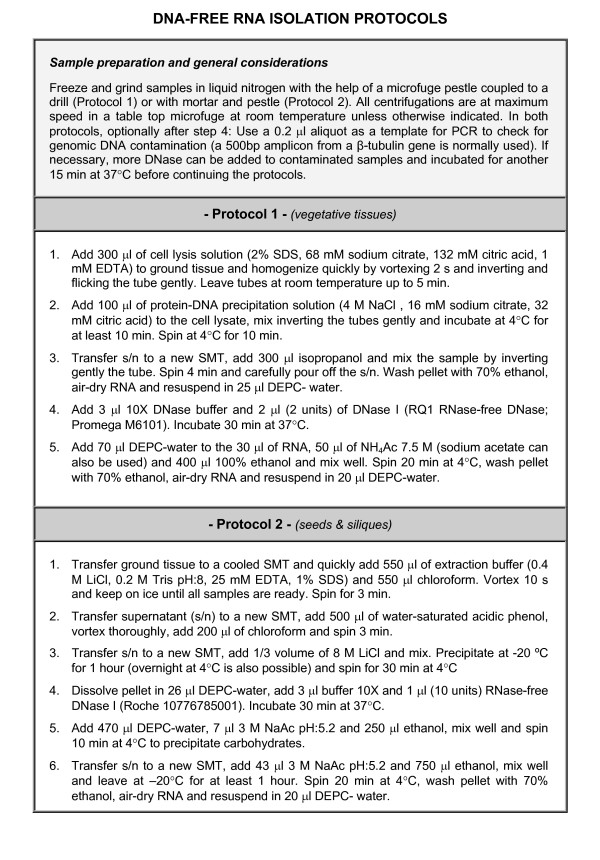
**DNA-free RNA isolation protocols**. Several steps common for both protocols are summarized at the top of the figure. General considerations taken when working with RNA, such as glassware sterilization or wearing gloves, are also applicable to these protocols.

**Figure 2:**
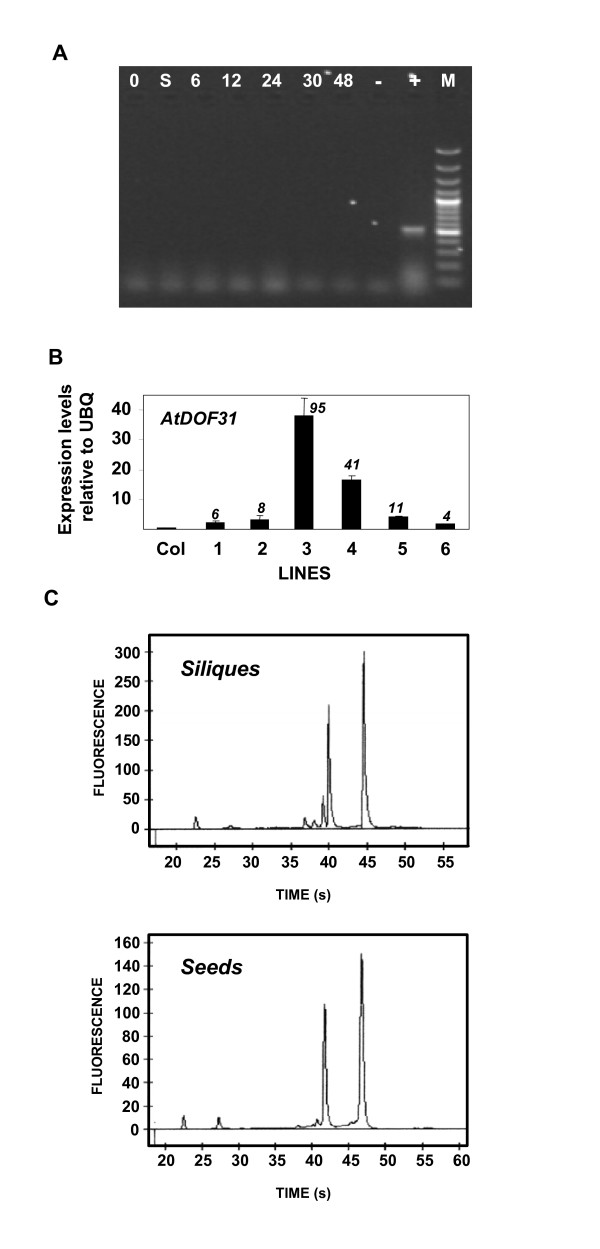
**PCR to detect gDNA, RNA electropherograms and RT-qPCR on Arabidopsis seeds and leaves**. (A) PCR products after 35 cycles with β-*tubulin *specific oligonucleotides on DNase treated total RNA isolated from dry (0), stratified (S) and germinating seeds (6 to 48 hours); (-): no template; (+): gDNA; (M): molecular marker. (B) qPCR with *AtDOF31 *specific oligos on cDNAs prepared from total RNA samples isolated from leaves of wild-type (Col-0) or transgenic plants overexpressing the *AtDOF31 *gene (lines 1 to 6). Expression levels relative to ubiquitin (UBQ; At5g25760) are shown and numbers in italic inside the graph indicates fold differences with Col-0. (C) Electropherograms of 500 ng total RNA samples from seeds and siliques (Agilent 2100 Bioanalyzer). Electrophoretic RNA migration time and fluorescence values correlate with RNA size and quantity, respectively, and are calculated by comparison to a RNA ladder (Agilent Technologies, Palo Alto, CA, USA).

The protocol presented here (Figure 1 – Protocol 1) is a modification of United States Patent 5973137 [6] and has been adapted to suit RT-qPCR. We routinely use 20–30 mg of Arabidopsis tissues although as little as 5–10 mg can be used. If more tissue is required, scaling up the volumes is advisable. The yield depends on the tissue and typically varies between 10 to 30 μg of total RNA. It is fast (24 samples can be processed in about 60 minutes), inexpensive, robust and buffers are free of toxic organic solvents and stable at room temperature. It uses an anionic detergent and a chelator at low pH to solubilize cellular components and inactivate RNases. This is followed by an RNA purification step using a high concentration, low pH salt reagent to reduce significantly contaminating DNA and proteins and a short isopropanol precipitation. At this point, the isolated samples are not completely pure due to the coprecipitation of some debris and salts, but they are pure enough to be treated with DNase. We have found that 30 min incubation at 37°C with 2 units of DNase (RQ1 RNase-free DNase; Promega M6101) is enough to reduce gDNA contamination below PCR detection levels. However, if gDNA contamination is detected in a given sample, 2 more units of DNase can be added and the sample incubated at 37°C for another 15 min. It is advisable to check for gDNA contamination by performing PCR reactions on the RNA samples before the final RNA cleaning step. We use 1/125 sample volume (0.2 μl) as a template for the PCR since the same fraction will be used later from the synthesized cDNA for the qPCR. Figure 2A shows that gDNA could not be detected after 35 cycles of PCR on RNA samples. If the samples are free of gDNA, the RNAs are purified by ethanol precipitation with ammonium acetate to avoid coprecipitation of dNTPs generated by the DNase. RNA absorbance (A_260_/A_230 _and A_260_/A_280_) values (always above 2.0) and the graphs obtained after plotting RNA species versus quantity (electropherograms; Agilent 2100 Bioanalyzer) indicated the absence of undesirable contaminants (data not shown).

To demonstrate the quality of the samples and sensitivity of the protocol, RT-qPCR was performed on RNA samples isolated from Arabidopsis leaves of the Columbia-0 ecotype (wild type) and leaves of transgenic plants overexpressing *AtDOF31 *(*35S:AtDOF31*). *AtDOF31 *is a DOF transcription factor (TF) with very low relative transcript abundance, whose expression cannot be detected by *in situ *hybridizations or by promoter::GUS fusions (Oñate-Sánchez et al., unpublished). Gene-specific oligonucleotides were chosen to amplify regions near the 5' end to ensure RNA integrity and cDNA synthesis efficiency and a ubiquitin gene was amplified for sample normalization. cDNA was synthesized according to [7], diluted 10 times and 2 μl were used as a template for real time PCR together with forward and reverse oligonucleotides (0.5 μM each) in 1× Power SYBR Green Master mix (Applied Biosystems). Cycling conditions (Applied Biosystems 7300 instrument) were as follows: 10 min at 95°C, 50 cycles of 15 s at 95°C and 60 s at 60°C, linked to a default dissociation stage program to detect non-specific amplification. As shown in Figure 2B, basal levels of *AtDOF31 *expression were detected in the wild type sample and several transgenic lines with different levels of *AtDOF31 *overexpression relative to the control (Col-0) were identified.

### RNA extraction from Arabidopsis seeds and siliques

Arabidopsis seeds and siliques are tissues with a high content of polysaccharides and other compounds, which can appear as contaminants of RNA samples [2] and attempts to isolate clean RNA from these tissues using the protocol described above were unsuccessful. Although there are published protocols that use organic extractions [1-5], we have improved them by using simple buffers and including a step to precipitate carbohydrates, which bypass the need to use high salt concentrations in the initial steps of purification (Figure 1 – Protocol 2). We have also shortened the protocol and slightly increased yield, despite starting from smaller amounts of tissue. Due to the small size of Arabidopsis seeds, the availability of sufficient quantities of starting material is a limiting factor, especially when working with conditions or genotypes producing low seed set. Published protocols on seed RNA isolation use 50 mg or higher amounts of tissue and/or non-SMTs at some point of the procedure [2,5]. Higher RNA yields (1,584 ± 304 μg/g) than published protocols were obtained using only 20 mg of seeds. We observed that using phenol directly over the extraction buffer produced brownish RNA pellets, probably due to the presence of phenolic compounds. We decided to use chloroform prior to phenol to remove the phenolic compounds [[5] and references therein]. To shorten the protocol we mixed the extraction buffer with chloroform, thus avoiding RNA degradation and removing the phenolic compounds at the same time. The aqueous phase was then extracted with acid phenol to remove proteins and as much DNA as possible, and chloroform was added in the same step to facilitate the separation of organic and aqueous phases and to eliminate any possible contamination with phenol. Genomic DNA contamination was removed after RNA was precipitated with ClLi (data not shown). Protein and salts from the DNase digestion were removed during the final RNA purification step, which was divided into two parts to precipitate firstly, carbohydrates with low concentrations of sodium acetate and ethanol and secondly, RNA by increasing those amounts up to standard concentrations. RNA absorbance (A_260_/A_230 _and A_260_/A_280_) values (always above 2.0) and their electropherograms indicated the absence of undesirable contaminants (Figure 2C).

**Figure 3:**
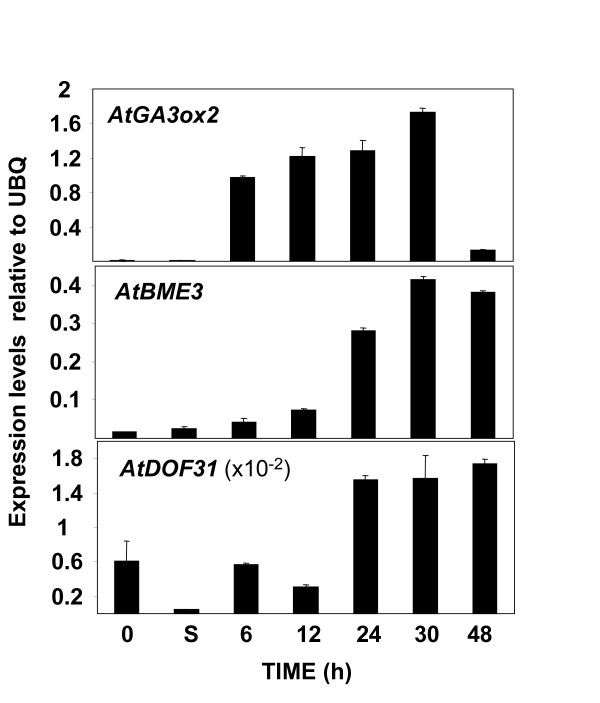
**qPCR on selected genes to determine protocol 2 specificity and sensitivity**. qPCR with gene (indicated inside the graph) specific oligonucleotides on cDNAs prepared from total RNAs described in Figure 1A. Expression levels relative to ubiquitin (UBQ; At5g25760) are shown.

As in the first protocol, we synthesized cDNA from the isolated RNAs and performed qPCR using gene-specific oligonucleotides to amplify the *AtDOF31 *gene as well as previously characterized genes with different expression levels and kinetics during Arabidopsis seed development and germination. *AtGA3ox2 *(At1g80340) is expressed predominantly in the cortex and endodermis of the embryonic axis in germinating Arabidopsis seeds [8] and *AtBME3 *(At3g54810) encodes a GATA-type zinc finger TF gene whose promoter is weakly expressed only at the micropylar end of the embryonic axis during germination [9]. Figure 3 shows that *AtDOF31 *is significantly induced during seed germination suggesting it may be an important player in the regulation of this seed phase since it is expressed at low levels outside this process. The kinetics observed for the *AtGA3ox2 *and *AtBME3 *genes (Figure 3) were in agreement with published results and expression kinetics for the seed-specific cruciferin 3 gene (early and mid-maturation phases) and the late embryogenesis abundant 76 gene (later stages of maturation) during seed development (data not shown) matched those described previously [10,11]. All these data confirm the suitability of this protocol.

## Discussion

We have described two simplified, inexpensive and robust protocols to isolate DNA-free RNA from Arabidopsis tissues. As far as we know, all non-commercial protocols for RNA isolation from plant tissues use organic solvents during the first steps. We present here a protocol that uses two simple buffers without organic solvents that includes a 30 min DNase digestion and requires only 90 min to obtain 24 DNA-free RNA samples. Although only data from leaves is presented here, RNA from most Arabidopsis tissues can be isolated since we have successfully used it with stems, roots and flowers (data not shown) and also from different vegetative tissues of the legume model plant *Medicago truncatula *(Gao, personal communication). The only tissues not amenable for the previous protocol were seeds and siliques and we streamlined and improved existing protocols that use organic extractions for RNA purification from those recalcitrant tissues. This protocol has also been successfully used in the isolation of RNAs from developing barley seeds (Oñate-Sánchez and Vicente-Carbajosa, unpublished). Altogether, our results indicate that the use of these protocols can be extended to other plant species.

We determined that the RNA obtained at an intermediate step of the isolation protocol was of sufficient quality to allow DNase digestion before the final purification steps. This modification shortens the protocol and maximizes yield bypassing the need to double precipitate the RNA. RNA integrity and purity are key factors influencing cDNA synthesis and ultimately detection and accurate quantification of RNAs, especially low abundance RNAs such as those derived from many TF coding genes. *AtDOF31 *and *AtBME3 *are expressed at low levels and *AtBME3 *mRNA has a length (1700 bp) above the average for a TF. After cDNA synthesis using oligo dT and qPCR using specific oligonucleotides annealing as far as possible from the polyA, we were able to detect and quantify the expression levels of both genes in all samples analyzed, confirming sensitivity and specificity.

Accessibility to these protocols is ensured by its simplicity and low cost of the materials used. With the increase of genomic approaches involving the manipulation of large numbers of samples, we feel these protocols will be extremely useful for plant scientists.

## Competing interest

The authors declare that they have no competing interests.

## Authors' contributions

LOS was responsible for experiments and supervision of this study and JVC contributed to the supervision.
